# Valuing health-related quality of life in heart failure: a systematic review of methods to derive quality-adjusted life years (QALYs) in trial-based cost–utility analyses

**DOI:** 10.1007/s10741-019-09780-7

**Published:** 2019-03-22

**Authors:** Jenny Rankin, Donna Rowen, Amanda Howe, John G. F. Cleland, Jennifer A. Whitty

**Affiliations:** 1 Health Economics Group, Norwich Medical School, University of East Anglia, Norwich Research Park, Norwich, NR4 7JT UK; 2 School of Health and Related Research, University of Sheffield, Sheffield, UK; 3 Robertson Centre for Biostatistics & Clinical Trials, University of Glasgow & National Heart & Lung Institute, Royal Brompton & Harefield Hospitals, Imperial College, London, UK

**Keywords:** Heart failure, Cost utility analysis, Economic evaluation, Cost-effectiveness, QALY, Health-related quality of life

## Abstract

The accurate measurement of health-related quality of life (HRQoL) and the value of improving it for patients are essential for deriving quality-adjusted life years (QALYs) to inform treatment choice and resource allocation. The objective of this review was to identify and describe the approaches used to measure and value change in HRQoL in trial-based economic evaluations of heart failure interventions which derive QALYs as an outcome. Three databases (PubMed, CINAHL, Cochrane) were systematically searched. Twenty studies reporting economic evaluations based on 18 individual trials were identified. Most studies (*n* = 17) utilised generic preference-based measures to describe HRQoL and derive QALYs, commonly the EQ-5D-3L. Of these, three studies (from the same trial) also used mapping from a condition-specific to a generic measure. The remaining three studies used patients’ direct valuation of their own health or physician-reported outcomes to derive QALYs. Only 7 of the 20 studies reported significant incremental QALY gains. Most interventions were reported as being likely to be cost-effective at specified willingness to pay thresholds. The substantial variation in the approach applied to derive QALYs in the measurement of and value attributed to HRQoL in heart failure requires further investigation.

## Introduction

Heart failure (HF) is common and costly to manage; it accounts for 1–3% of health care expenditure in Western Europe, North America and Latin America and causes or complicates about 5% of all US and European hospital admissions amongst adults [1]. The costs, prevalence and complexity of treating HF are increasing, along with ageing of the population. Identifying and supporting patient access to interventions that are both clinically and cost-effective are required to optimise the use of resources.

Health-related quality of life (HRQoL) is an important outcome measure in HF that is influenced by physical, emotional or social function, role performance, pain and fatigue. There has been a drive towards patient-reported outcome measures (PROMs) and patient-reported experience measures (PREMs) in health care systems, including the British NHS. Thus, the accurate measurement and valuation of HRQoL and its response to therapy are essential for choosing treatments and allocating resources. When considering the cost-effectiveness of interventions, the quality-adjusted life year (QALY) is the outcome of choice for most decision-making bodies (such as the National Institute for Health and Care Excellence, NICE in the UK [2]), as it accounts for both HRQoL and survival, and their changes, in a single metric. QALYs are typically obtained from generic preference-based measures (PBMs) such as the EQ-5D (three- or five-level version) to provide utility values and these are multiplied by the duration lived in a health state. PBMs describe HRQoL as a series of health states and then assign a utility weight to each health state on a common scale, according to the preferences of members of the public for being in different health states. An alternative method for generating utility values is the direct elicitation of utilities from the patients themselves, using valuation methods such as the time trade-off (TTO), standard gamble (SG) or discrete choice experiment (DCE). QALY gains can subsequently be compared between interventions for use in economic evaluation.

Accurate measurement and valuation of HRQoL relies on the availability of a PBM that is sensitive to change. Generic PBMs are commonly used in economic evaluation, and there is evidence to support the validity and reliability of the commonly used EQ-5D-3L in cardiovascular disease, particularly in moderate to severe health states [3]. However, generic measures may lack sensitivity to change as they do not capture important symptoms of HF such as breathlessness, loss of self-control and tiredness [4, 5]. Condition-specific measures of HRQoL such as the Minnesota Living With Heart Failure (MLWHF) questionnaire and Kansas City Cardiomyopathy Questionnaire (KCCQ) capture these symptoms. The KCCQ has been reported to be more sensitive than the generic EQ-5D-3L and Short-Form Survey (SF)-12 measures, particularly for detecting small rather than large changes in disease severity [3, 4, 6, 7]. Consequently, researchers have called for the inclusion of condition-specific measure alongside generic measures when capturing effectiveness for these conditions [8].

Whilst condition-specific measures are sensitive in capturing HRQoL, none of the available condition-specific measures in HF are preference-based [9]. Therefore, they cannot be used directly to generate QALYs. Other approaches such as mapping have been used to generate utility weights where no PBM was used, meaning a HF-specific measure could be used to measure HRQoL and this could be mapped to a measure such as the EQ-5D to generate utility weights. However, mapping is only appropriate if both measures are appropriate for the patient population, and relies on overlap between the two measures. Any symptoms captured in the HF-specific measure are unlikely to feature in the mapping model, meaning the sensitivity of the HF-specific measure to change is not necessarily maintained when mapped to EQ-5D. Therefore, the lack of availability of utility indices for condition-specific measures is likely to limit their use in the economic evaluation of HF interventions.

Accordingly, we conducted a systematic review to identify and describe the approaches used to measure and value change in HRQoL in trial-based economic evaluations of HF interventions which derive QALYs as an outcome measure. We sought to investigate the extent to which utility weights are generated using different approaches: generic PBM, mapping to a PBM or directly ascertained using a valuation method. A secondary objective was to identify whether published papers reported whether interventions for HF were cost-effective.

## Methods

### Protocol and registration

The review protocol is registered at the International Prospective Register of Systematic Reviews (PROSPERO) and can be accessed at: https://www.crd.york.ac.uk/prospero/display_record.php? RecordID = 78519 registration number CRD42017078519.

### Eligibility criteria

The inclusion and exclusion criteria are presented in Table 1. Randomised controlled trials that were published in full, in English, and compared costs and benefits expressed as QALYs as an outcome measure were included if they evaluated an intervention designed to investigate the treatment or management of HF in adults (≥ 18 years old). There were no upper age limit, sex or publication date restrictions. Studies that included participants without HF, systematic reviews, modelled studies, meta-analyses and those published as abstract only were excluded.

**Table 1:** Inclusion and exclusion criteria

Inclusion criteria (if all of the following met)	Exclusion criteria (if any of the following met)
1. Original research	8. Papers other than in the English Language
2. Adults (aged 18 and over) of any sex or ethnic group	9. Design/protocol papers, systematic reviews, meta-analyses and commentaries/editorials
3. Interventions designed to treat or manage heart failure	10. Effectiveness estimates not based on actual trial data (e.g. hypothetical intervention or summarised effect)
4. Trial-based analyses based on data from randomised control trials	11. Trials investigating conditions other than heart failure
5. Comparison of costs and benefits expressed as QALYs	12. Studies published as abstract only
6. Published papers in English	13. Prevention and diagnostic interventions
7. Participants with heart failure	14. Model-based studies

### Search strategy

PubMed Central, Cumulative Index to Nursing and Allied Health Literature (CINAHL) and the Cochrane Library (NHS Economic Evaluation Database) were searched between 26 June and 3 July 2017, with no date restrictions.

The following search terms were used:

- PubMed Central: “heart failure”[Abstract] AND (cost utility analysis [Abstract] OR CUA [Abstract] OR economic evaluation [Abstract] OR cost effectiveness [Abstract])
- Cochrane Library: “heart failure” AND (cost utility analysis OR CUA OR economic evaluation OR cost effectiveness)
- CINAHL: “heart failure” AND (cost utility analysis OR CUA OR economic evaluation OR cost effectiveness)

### Study selection

The results from the databases were combined, and duplicates were removed. Papers were screened on their title and abstract. Potentially relevant papers were retrieved in full and screened against the inclusion/exclusion criteria. Bibliographies of relevant papers were hand-searched for any sources potentially missed within the database searches.

### Data extraction

The characteristics and results of relevant studies were extracted based on an amended version of a standardised data collection form recommended by the Cochrane Training (Cochrane Library: http://training.cochrane.org/resource/data-collection-forms-intervention-reviews). The form was amended to collect characteristics relevant to describe the economic evaluation and HRQoL measure and valuation.

The following were extracted:

- Measurement and valuation of HRQoL including measure and approach used to generate utility weights
- The elicitation method, tariff and population used to derive the utility weights used to value HRQoL
- The mean difference in QALYs between the intervention and control groups from baseline to follow-up with significance levels and confidence intervals (if available)
- Incremental cost per QALY of intervention(s) and indication of the level of uncertainty (such as confidence interval) around that estimate
- Probability of cost-effectiveness at a specified threshold

Where a study cited other papers as the source of the utility weights to derive QALYs, the original source of utility weights was retrieved to enable a description of the approach to deriving QALYs.

### Quality assessment

The Consolidated Health Economic Evaluation Reporting Standards (CHEERS) statement was used to assess the reporting quality of studies [10]. Each of the 24 items in the CHEERS checklist was assigned a weight ranging from zero to two (representing studies that did not report, reported poorly or reported well) which were used to calculate an average reporting quality score.

## Results

### Study selection

**Fig. 1:**
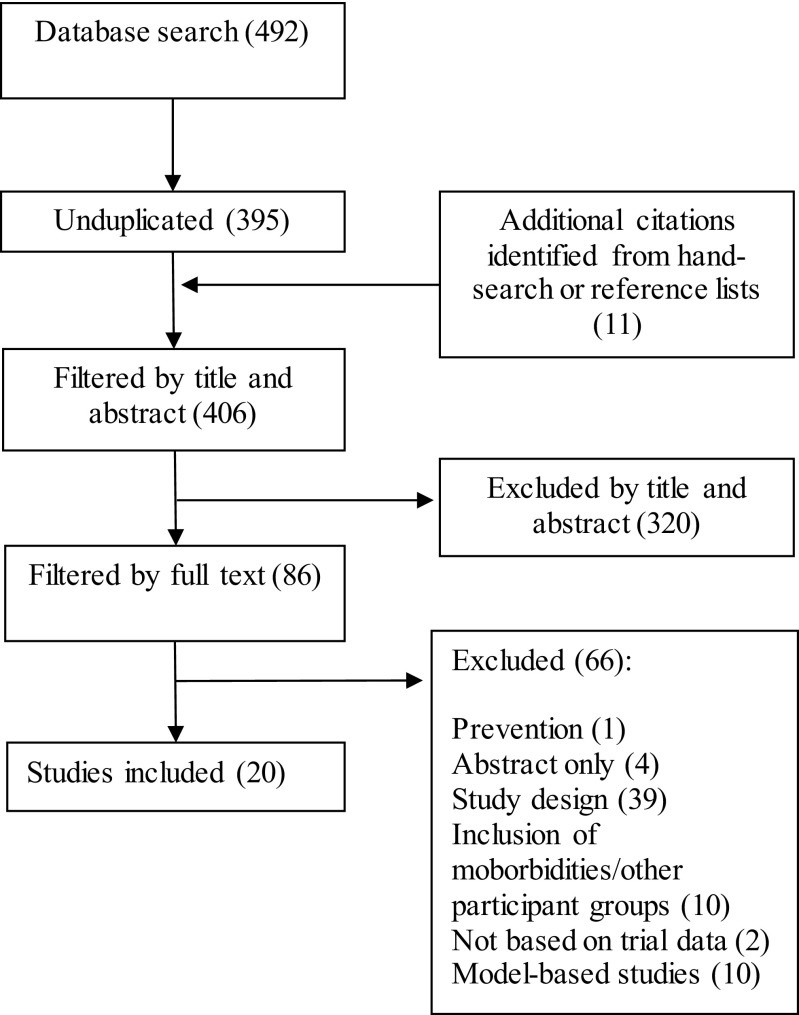
Study selection

A total of 492 citations were identified by the database searches, with 11 additional citations identified by hand searching (Fig. 1). Twenty studies were included in the final review (Table 2).

**Table 2:** Trial-based evaluations (*n* = 20 cost–utility analyses based on 18 trials)

			Study design			Study population		
Author and year	Data source	Setting; perspective	Intervention vs. control	Discount rate	Sample size (*n*)	Age (mean)	Sex (males %)	NYHA^†‡^ (%)
Agvall et al. (2014) [19]	The benefits of using a heart failure management programme in Swedish primary health care trial	Sweden; health care system^§^	Management program vs. usual care	NR	160	75	69.4	I (6) II (59) III (36)
Blomstrom et al. (2008) [11]	The Cardiac Resynchronisation in Heart Failure trial (CARE-HF)	Europe; Denmark, Finland and Sweden; health care system	Pharmacological therapy (PT) with cardiac resynchronisation therapy vs. PT alone	3%	813	I 67* C 66	73.4	III (87) IV (13)
Boyne et al. (2013) [20]	Telemonitoring in Heart Failure trial (TEHAF)	The Netherlands; health care system	Telemonitoring vs. usual care	0%	382	71	59	II (57) III (40) IV (3)
Calvert et al. (2005) [12]	CARE-HF	Europe; UK health care system	Pharmacological therapy (PT) with cardiac resynchronisation therapy vs. PT alone	3.5%	813	I 67* C 66	73.4	III (87) IV (13)
Capomolla et al. (2002) [15]	Own trial	Italy; societal	Day-hospital management vs. usual care	5%	234	56	83.8	I-II (65) III-IV (35)
Cui et al. (2013) [27]	Testing the Effectiveness of Health Lines in Chronic Disease Management of Congestive Heart Failure trial (Health Lines)	Canada; health care system	Health Lines (I1) and Health Lines and in-house monitoring (I2) vs. standard treatment	0%	179	75	52	II (22) III (47) IV (31)
Hansson et al. (2016) [16]	Person-Centred Care in Patients with Chronic Heart Failure trial (PCC-HF)	Sweden; health care system	Person-centred vs. conventional care	0%	248	I 77.5 C 80.3	58.9	I (5) II (35) III (54) IV (7)
Hebert et al. (2008) [21]	Own trial	US; societal and payer	Nurse-managed disease management vs. usual care	NR	406	59.4	54	I (18) II (22) III (14) IV (45)
Maniadakis et al. (2011) [13]	CARE-HF	Europe; Greek health care system	Pharmacological therapy (PT) with cardiac resynchronisation therapy vs. PT alone	3%	813	I 67* C 66	73.4	III (87) IV (13)
Maru et al. (2015) [22]	Which Heart Failure Intervention Is Most Cost-Effective & Consumer Friendly in Reducing Hospital Care trial (WHICH)	Australia; health care system	Home vs. clinic based care	5%	280	71	73	II/III (85) IV (15)
Mejia et al. (2014) [23]	Nurse Facilitated Self-management Support for People with Heart Failure and their Family Carers trial (SEMAPHFOR)	UK; National Health Service	Nurse facilitated cognitive behavioural self-management programme vs. usual care	0%	260	70.6	72	II (68) III (30) IV (2)
Neumann et al. (2015) [24]	Interdisciplinary Network for Heart Failure trial (INH)	Germany; societal	Nurse-led management programme vs. usual care	NR	715	I 67.7 C 69.4	70.6	I (2) II (58) III (36) IV (4)
Patel et al. (2008) [25]	Own pilot trial	Sweden; health care system^§^	Home vs. conventional care	NR	31	I 77 C 78	67.7	II (3) III (94) IV (3)
Postmus et al. (2016) [17]	Coordinating Study Evaluating Outcomes of Advising and Counselling in Heart Failure trial (COACH)	The Netherlands; health services	Basic (I1) and intensive (I2) additional nurse support vs. usual care	NR	1023	71	62	II (50) III (46) IV (4)
Reed et al. (2010) [29]	Heart Failure: a Controlled Trial Investigating Outcomes of Exercise Training trial (HF-ACTION)	US, Canada and France; societal	Exercise training plus usual care vs. usual care	3%	2331	I 59.2 C 59.3	71.6	II (63) III (36) IV (1)
Reilly et al. (2015) [26]	Quality HF-diabetes trial	US; health care services	Self-care vs. usual care	0%	134	57.4	88	II (42) III (50) IV (8)
Sahlen et al. (2016) [18]	Palliative Advanced Home Care and Heart Failure Care trial (PREFER)	Sweden; health services provider	Palliative advanced home and heart failure care vs. usual care	NR	72	I 81.9 C 76.6	72.2	III (71) IV (29)
Sánchez et al. (2010) [31]	Own trial	Spain; hospital	Peritoneal dialysis vs. conservative therapy	NR	17	64	65	III (59) IV (41)
Sanders-van Wijk et al. (2013) [30]	Intensified vs. Standard Medical Therapy in Elderly Patients with Congestive Heart Failure trial (TIME-CHF)	Switzerland and Germany; third-party payer	N-Terminal Pro-B-Type natriuretic-guided therapy vs. symptom-guided therapy	0%	467	76	66	II (27) III-IV (73)
Zanaboni et al. (2013) [28]	Evolution of Management Strategies of Heart Failure Patients with Implantable Defibrillators trial (EVOLVO)	Italy; health care system and patient	Remote monitoring vs. conventional in-person evaluations	NR	200	I 66* C 69*	78.5	I (12) II (70) III (19)

*I* Intervention, *C* control, *I1* intervention 1, *I2* intervention 2, *NR* not reported

*Median

^†^Components may not add to 100 due to rounding

^‡^New York Heart Association classification (I to IV)

^§^Assumed as not explicitly reported in paper

### Study characteristics

The 20 papers included within the review were trial-based evaluations based on data sourced from 18 individual randomised controlled trials (RCT), with three papers [11–13] based on the Cardiac Resynchronisation in Heart Failure (CARE-HF) trial [14]. The date of publishing ranged from 2002 [15] to 2016 [16–18].

The majority of papers (*n* = 16) focused on management interventions as opposed to treatment (*n* = 4) for HF. Management interventions included the following: nurse led [17, 19–26], telemonitoring [27, 28], outpatient clinic based [15], person-centred care [16, 18], exercise training [29] and NT-proBNP-guided therapy [30]. Treatment interventions included cardiac resynchronisation therapy [11–13] and peritoneal dialysis [31].

Most papers adopted the perspective of their respective countries’ health care system or third-party payers, and one study [28] included the costs and effects from a patient perspective. The remaining studies adopted a societal [15, 24, 29], a combination of both a societal and payer [21] or a single hospital perspective [31]. The study perspective was not explicitly reported in two papers [19, 25]; a health care system perspective was assumed in both because of the costings involved within the trials.

Sample sizes within the trials ranged from 17 [31] to 2331 [29] with a total of 7952 participants across all studies (accounting for the individuals in the CARE-HF trial only once). The overall population studied within the trials was predominantly men (68.2%) with mean/median ages ranging from 56 ± 10 [15] to 81.9 ± 7.2 years [18]. Fifteen papers reported average participant ages of > 60 years. Three papers excluded participants based on age: those aged under 21 (with an age limit of 82) [26], under 40 [27] and under 60 years [30].

New York Heart Association (NYHA) functional class, a commonly used physician-assessed tool measuring patient’s functional ability in HF, was reported in all papers reviewed. Seventeen trials reported individual participant category numbers for each NYHA class (NYHA classes were grouped in three studies [15, 22, 30] and therefore were not included in the following summary). Most participants were assigned to NYHA class II (47.5%) and III (43.7%) rather than class IV (the most severe; 6.9%) or class I (asymptomatic; 1.9%). Overall, eight reports had exclusion criteria in relation to participant NYHA class, with five studies excluding NYHA class I [22, 25, 26, 29, 30] and CARE-HF (three reports) excluded NYHA class I and II [11–13].

The discount rate used for assessing costs and effects in the trial-based economic evaluations ranged from to 0% [16, 20, 23, 26, 27, 30] in the studies who had a follow-up of less than 12 months to 3% [11, 13, 29], 3.5% [12] and 5% [15, 22]. No discount rate was reported in eight studies [17–19, 21, 24, 25, 28, 31].

### Measurement and valuation of HRQoL to derive QALYs

A summary of the approach used to derive QALYs in the base-case analyses for the 20 studies is provided in Table 3.

**Table 3:** Methods to derive QALYs applied in the studies (20 cost–utility analyses)

Author and year	Measure of HRQoL collected in trial	Valuation method used to assign utility weights	Follow-up	Mean difference between groups at follow-up (QALYs per person) (95% CI) significance
Agvall et al. (2014) [19]	NYHA class	Published study in which patients valued own health using TTO, categorised by NYHA class [32]	Baseline and 12 months	− 0.01 *NS*
Blomstrom et al. (2008) [11]	EQ-5D-3L^†^	UK weights (TTO) [33]	Within trial: Baseline, 3 months	Within trial 0.20 Extrapolated 0.91 *NR*
	MLWHF	MLWHF mapped to EQ-5D-3L [12]	Extrapolated beyond trial: 18 months and end of study (mean follow-up 29.4 months)	
Boyne et al. (2013) [20]	EQ-5D-3L	UK weights (TTO) [33]	Baseline, 3, 6 and 12 months	− 0.0031 (− 0.0552 to 0.0578) *NS*
Calvert et al. (2005) [12]	EQ-5D-3L^†^	UK weights (TTO) [33]	Within trial: Baseline, 3 months	Extrapolated 0.22 (0.13 to 0.32)^#^
	MLWHF	MLWHF mapped to EQ-5D-3L	Extrapolated: 18 months and end of study (mean follow-up 29.4 months)	
Capomolla et al. (2002) [15]	Own health state	Patients in trial used TTO to value own health state	12 months	0.080 *p < 0.01*
Cui et al. (2013) [27]	SF-36	Converted into SF-6D UK weights (SG)^‡^ [34]	Baseline, 3 (6 and 12) months*	I2 0.04 *NR*
Hansson et al. (2016) [16]	EQ-5D-3L	UK weights (TTO) [33]	Baseline and 3 months	0.001 *NR*
Hebert et al. (2008) [21]	SF-12	Mapped to HUI-3 [35]	Baseline, 3, 6, 9 and 12 months	0.0497 (0.0054 to 0.0940)^#^
		Mapped to EQ-5D-3L [35]		0.0430 (0.0012 to 0.0848)^#^
Maniadakis et al. (2011) [13]	EQ-5D-3L^†^	UK weights (TTO) [33]	Within trial: 3 months	Extrapolated 1.41 Within trial 0.20 *NR*
	MLWHF	MLWHF mapped to EQ-5D-3L [12]	Extrapolated: 18 months and end of study	
Maru et al. (2015) [22]	EQ-5D-3L	Australian weights (TTO) [36]	Baseline and 6, 12, 18 months, extrapolated to extended follow-up (median 3.2 years)	0.26 (− 0.03 to 0.56) *p = 0.078*
Mejia et al. (2014) [23]	EQ-5D-3L	UK weights (TTO) [33]	Baseline, 3, 6 and 12 months	− 0.02 (− 0.09 to 0.05) *p = 0.537*
Neumann et al. (2015) [24]	EQ-5D (3 or 5-level NR)	German weights (source NR)	Baseline and 6 months	0.022 *p = 0.02*
Patel et al. (2008) [25]	EQ-5D-3L (VAS) Own health state	No tariff, i.e. VAS for own health state SG to value own health state	Baseline and 12 months	0.07 *NS* 0.01 *NS*
Postmus et al. (2016) [17]	SF-36	Converted into SF-6D UK weights (SG)^‡^ [34]	Baseline, 1, 6, 12 and 18 months	I1 0.023 I2 0.004 *NR*
Reed et al. (2010) [29]	EQ-5D-3L	US weights (TTO)	Baseline, quarterly through first year and annually at final visit (max. 4 years)	0.03 (− 0.06 to 0.11) *NS*
Reilly et al. (2015) [26]	EQ-5D-3L	US weights (TTO) [37]	Baseline and 6 months	0.04 (− 0.04 to 0.11) *NS*
Sahlen et al. (2016) [18]	EQ-5D-3L	Tariff NR	Baseline and 6 months (± 2 weeks)	0.03 *p = 0.026*
Sánchez et al. (2010) [31]	EQ-5D-3L	Methods unclear, appears to be TTO (Spain)	Baseline and 6 months	0.227 (utility^§^) *p < 0.01*
Sanders-van Wijk et al. (2013) [30]	SF-12	Converted into SF-6D [34]	Baseline, 12 and 18 months	0.05 (− 0.02 to 0.11) *p = 0.35*
Zanaboni et al. (2013) [28]	EQ-5D (3L assumed)	European EQ-net (VAS) [38]	Baseline and 16 months	0.065 *p = 0.03*

Italicised entries indicate signifiance level

*NR* not reported, *I* intervention, *C* control, *NS* not significant, *I1* intervention 1, *I2* intervention 2, *I3* intervention 3, *MLWHF* Minnesota Living With Heart Failure questionnaire, *TTO* time trade-off, *SG* standard gamble, *VAS* visual analogue scale, *EQ-5D* EuroQol, *SF-36/SF-12* Short-Form Survey 36/12, *NYHA* New York Heart Association functional classification

*QALYs reported on results from first survey (3 months)

^†^Utilities at 18 months and end of study were estimated from MLWHF scores collected at 18 months and end of study on the basis of a mixed model of the relationship between change in EQ-5D-3L score to change in MLWHF

^‡^Assumption of UK Weights (SG) use

^§^QALYs calculated but utility weights reported only

^#^Confidence interval suggest significant but *p*-value not reported

### Description of health states used to derive QALYs

Most (*n* = 17) of the studies used a validated, generic PBM of HRQoL completed by participants to describe the health status of participants. The EQ-5D was the most commonly used (*n* = 13), with most studies using the 3L version. Two studies did not indicate the version of EQ-5D used (3L or 5L) [24, 28] but seem likely to have used the 3L version based on publication date (2013 and 2015). Two studies used SF-36, and two studies used SF-12 to describe participants’ health states. Three of the 17 studies which used a generic PBM also used the MLWHF condition-specific questionnaire to collect longer term (median 29.4 month) follow-up data to describe HRQoL for the purpose of deriving QALYs in a cost–utility analysis that extended outcomes beyond the end of the initial trial. These three cost-utility analyses (CUAs) were all undertaken alongside the CARE-HF trial [11–13]. Of the remaining three studies, two studies used patients own perceived health states to derive QALYs [15, 25], one of which also described health status using the EQ-5D visual analogue scale (VAS) [25]. The final study [19] used the physician-reported NYHA assessment to describe health status [32].

### Valuation of HRQoL

Of the 17 studies using a generic PBM to describe health status of the patients in their trials, most (*n* = 13) reported that they applied an existing utility tariff for valuation. For the 13 studies using EQ-5D, eight studies applied the UK tariff in which the EQ-5D-3L health states were valued by a sample of the public using TTO methods, one study applied Australian TTO weights, and one study applied the European EQ-net weights, in which health states were valued using VAS methods. The remaining three studies using EQ-5D to describe health status did not clearly report the method for deriving utilities [18, 24, 31]; although, in two studies, the German and Spanish tariffs based on TTO methods would appear to have been used to assign utility weights to the EQ-5D health states [24, 31]. Both studies using the SF-36 and one of the studies using the SF-12 to describe participants’ health status applied the SF-6D algorithm to assign utilities. The SF-6D algorithm was developed based on the preferences of a UK public sample for being in different health states, using the SG valuation method [34]. The final study using the SF-12 to measure participant health status [21] mapped SF-12 results onto the Health Utilities Index Mark 3 (HUI-3) and the EQ-5D-3L in two separate base case analyses using a conversion formula based on the results from a low-income minority population [35].

The three studies which used the MLWHF questionnaire, a condition-specific HRQoL instrument for which there is no utility tariff available, to both describe and value HRQoL alongside the EQ-5D-3L, were CUAs undertaken alongside the CARE-HF trial [11–13]. They used the MLHF data collected at a median of 29.4 months follow-up to model utility outcomes beyond the initial period of the trial. The authors assigned utility weights to model utility outcomes beyond the initial period of the trial based on a mixed model mapping the relationship between change in EQ-5D-3L and change in MLWHF which were both completed at baseline and 90 days follow-up in the trial [12].

Of the remaining three studies, two studies used patients’ direct valuation of their own health states to assign utility weights (one using the TTO approach [15] and one using the EQ-5D VAS and SG approaches [25]). The final study in which participants health status was described using NYHA [19] assigned utility weights to NYHA classes using a published study in which elderly patients with heart failure valued their own health status using TTO methods [32].

### Change in QALY reported by the studies

We attempted to examine whether the evaluations undertaken alongside trials identified significant changes in QALYs (Table 3). Only seven of the 20 analyses undertaken alongside trials reported significant incremental QALY gains, ranging between 0.022 and 0.22 QALYs per person over follow-up periods of up to a mean of 29.4 months. Interestingly, none of these generated utility weights using the combination of EQ-5D-3L with the UK TTO tariff, despite this being the most common approach to generate utility weights across the studies. Instead, the seven studies [12, 15, 18, 21, 24, 28, 31] reporting significant QALY differences used the EQ-5D-3L with US, Spanish or European utility weights, the EQ-5D (3L/5L not stated) with German weights, the MLWHF mapped to EQ-5D-3L, SF-12 mapped to EQ-5D/HUI-3 or direct TTO valuation by patients to derive QALYs. Eight analyses [19, 20, 22, 23, 25, 26, 29, 30] did not report significant QALY differences between interventions. Two of these used the EQ-5D-3L to describe health states combined with the UK tariffs, others used US (*n* = 2) or Australian (*n* = 1) tariffs to value the gain, one used the SF-12 converted to SF-6D utility weights, one used the EQ-5D-3L VAS scale to derive patient’s direct valuations for health states and another used NYHA class with utility weights derived from TTO from previous literature. Cost–utility analyses of five trials [11, 13, 16, 17, 27] did not report the significance of any change in QALY.

### Cost-effectiveness of interventions

Table 4 summarises the overall findings of the 20 studies with respect to the cost-effectiveness of interventions evaluated for the management or treatment of heart failure. Overall, most interventions were reported as being cost-effective using the thresholds the studies applied to their own evaluations (which were dependent upon country of study and relevant international agency). The incremental cost-effectiveness ratios (ICERs) ranged from a cost-saving of − 61,081 € [31] to 98,000 € [16] per QALY gained. Probabilities of being cost-effective ranged from 0.08 at a 20,000 € threshold [39] to around 1.0 at a 25,000 € threshold [13]. Eight interventions were reported as being dominant [17, 18, 22, 26, 28–31] compared to the control group, and one was reported as being dominated [23]. Two studies did not publish cost per QALY, and seven studies did not publish a cost-effectiveness probability estimate [15, 16, 18, 19, 25, 28, 31].

**Table 4:** Cost-effectiveness results of interventions (*n* = 20)

Author and year	Intervention vs. control	Results		
		Cost per QALY	Cost-effective probability	Quality (CHEERS) (%)
Agvall et al. (2014) [19]	Management program vs. usual care	NR	NR	72.9
Blomstrom et al. (2008) [11]	Pharmacological therapy (PT) with cardiac resynchronisation therapy vs. PT alone	Denmark 4759 € (1553 € to 12,637 €), Finland 3571 € (1169 € to 10,153 €), Sweden 6493 € (2669 € to 17,482 €)	99% at 50,000 € threshold	77.1
Boyne et al. (2013) [20]	Telemonitoring vs. usual care	40,321 €	48% at 50,000 € threshold	81.3
Calvert et al. (2005) [12]	Pharmacological therapy (PT) with cardiac resynchronisation therapy vs. PT alone	19,319 € (5482 € to 45,402 €)	83% at 29,400 € threshold	93.8
Capomolla et al. (2002) [15]	Day-hospital management vs. usual care	US$19,462 ($13,904 to $34,048)	NR	75.0
Cui et al. (2013) [27]	Health lines (I1) and Health lines and in-house monitoring (I2) vs. standard treatment	CAD$2975*	85.8% at $50,000 threshold	85.4
Hansson et al. (2016) [16]	Person-centred vs. conventional care	98,000 €	NR	91.7
Hebert et al. (2008) [21]	Nurse-managed disease management vs. usual care	US$17,543 (−$139,295 to $458,900) for EQ-5D conversion and US$15,169 (−$114,748 to $282,657) for HUI-3	64% at $50,000 threshold	93.8
Maniadakis et al. (2011) [13]	Pharmacological therapy (PT) with cardiac resynchronisation therapy vs. PT alone	6045 € (4292 € to 9411 €)	~100% at 25,000 € threshold	91.7
Maru et al. (2015) [22]	Home vs. clinic based care	Cost saving of AU$13,100 with an increase of 0.26 QALYs	96% at $20,000	91.7
Mejia et al. (2014) [23]	Nurse facilitated cognitive behavioural self-management programme vs. usual care	£69.49 per reduction of 0.004 (− 0.06 to 0.05) QALYs	45% at £20–30,000 threshold	97.9
Neumann et al. (2015) [24]	Nurse-led management programme vs. usual care	49,335 €	Between 55 and 90% up to 105,000 €	79.2
Patel et al. (2008) [25]	Home vs. conventional care	NR	NR	62.5
Postmus et al. (2016) [17]	Basic (I1) and intensive (I2) additional nurse support vs. usual care	Cost saving of 77 € for (I1) with QALY gain of 0.023 against usual care and cost saving 1178 € and I2 and 0.004 QALYs against (I2)	62% and 8% at 20,000 € threshold	72.9
Reed et al. (2010) [29]	Exercise training plus usual care vs. usual care	Cost saving of US$4300 with an increase of 0.03 QALYs	74.4% at $100,000 threshold	85.4
Reilly et al. (2015) [26]	Self-care vs. usual care	Cost saving of US$7647^‡^	79.3%^†^	75.0
Sahlen et al. (2016) [18]	Palliative advanced home and heart failure care vs. usual care	Cost saving of 1649 € and additional 0.25 QALYs	NR	87.5
Sánchez et al. (2010) [31]	Peritoneal dialysis vs. conservative therapy	− 61,081 €	NR	64.6
Sanders-van Wijk et al. (2013) [30]	N-terminal pro-B-type natriuretic-guided therapy vs. symptom-guided therapy	Cost saving of US$2979 and an increase of 0.05 QALYs	93% at $50,000 threshold	93.8
Zanaboni et al. (2013) [28]	Remote monitoring vs. conventional in-person evaluations	Cost saving of 888.10 € with additional 0.065 QALYs	NR	85.4

*NR* not reported, *I1* intervention 1, *I2* intervention 2, *I3* intervention 3

*ICER reported on results from first survey (3 months)

^†^Percent of samples falling in lower right quadrant of cost-effectiveness plane. No threshold reported

^‡^Cost analysis only

## Quality of reporting (CHEERS checklist)

Table 4 shows scores for the CHEERS checklist, reported as a percentage (%) out of a maximum score of 48. The quality of reporting of the studies ranged from 62.5% [25] (probably due to the small scale of this pilot study) to 97.9% [23].

## Discussion

Although HF is associated with a large health care burden, this systematic review identified rather few valid health economic analyses of relevant RCTs suggesting that many economic evaluations of HF interventions do not consider HRQoL as an outcome measure. For example, a search of the NHSEED (National Health Service Economic Evaluation) database which holds comprehensive records of published health economic evaluations identified 178 records with the term “heart failure” or “cardiac failure” in the title (database available at https://www.crd.york.ac.uk/CRDWeb/ search performed 4 January 2018). Moreover, Goehler et al. (2011) reviewed 34 decision-analytic modelled studies in HF, in which only 19 reported QALYs as an outcome measure. Nevertheless, most (though not all) of the cost–utility analyses identified in this review followed what is currently regarded as “best practice” for the derivation of QALYs and their consideration in economic evaluation [40, 41]. That is, they adopted generic measures, notably the EQ-5D-3L and SF-36 and its derivative (SF-12) on which participating patients describe their own health states, and then the preferences of a general population sample were used to value improvements in HRQoL by assigning utility weights from existing tariffs. The high frequency with which the EQ-5D-3L was used is consistent with the dominance of EQ-5D-3L in other clinical areas and the prescriptive guidance from NICE requiring EQ-5D-3L to generate utility weights in health technology assessments [2].

The methods used to derive QALYs in the identified studies were variable, however. In particular, they raise questions in the context of heart failure, around several methodological issues that are debated in the health state valuation literature. To undertake cost–utility analyses utilising the QALY as an outcome, it is necessary to both accurately measure change in HRQoL and to appropriately assign the utility value associated with that change using public preferences. Only generic PBMs which are designed to be used across a range of different conditions (such as the EQ-5D) are available as validated instruments to both measure and value HRQoL in HF. These have the advantage that if they are used consistently, they provide a common approach to measurement and valuation of HRQoL across all conditions, thus allowing a direct comparison of the benefit of allocating resources to heart failure alongside the benefit of allocating resources to address other health conditions. Condition-specific measures of HRQoL are more sensitive to change in HRQoL in HF, but existing instruments are not preference-based and so do not have a utility valuation tariff to derive QALYs [4, 6, 7, 42]. This might explain why only three cost–utility analyses (based on a single trial) attempted to use condition-specific HRQoL data to derive QALYs. The need for sensitive condition-specific measures to be used alongside generic measures to ensure the accurate capture and valuation of change in HRQoL has been raised previously across a range of clinical areas [5], including HF [43]. Indeed some international reimbursement agencies, such as NICE, allow the inclusion of analyses using condition-specific measures in sensitivity analyses to support the appraisal of health care interventions [2]. However, none of the studies identified in this review tested the impact on the cost-effectiveness estimates of using weights based on a condition-specific preference-based measure as opposed to a generic preference-based measure in the evaluation. Therefore, we are unable to provide further evidence to inform the debate on the relative value of generic vs. condition-specific measures in the setting of heart failure.

This review found mixed results in QALY outcomes; with similar numbers of studies finding significant, non-significant or unreported significance in differences between interventions in QALYs. This may be because the intervention was not effective, has a problem with trial size or design or the failure of the HRQoL tool to accurately capture change. Some measures used may not be responsive to changes in HRQoL. This would not be surprising as some trials failed to show improvements in HRQoL despite reductions in mortality and hospital readmission [44]. Alternatively, it may be that any change in HRQoL is captured, but the improvements in HRQoL are not considered meaningful according to the preferences of members of the public when they are valued and converted to QALYs. Within the review, a pilot study [25] used both the EQ-5D VAS and the SG to derive utility weights for the intervention and control groups in the same sample, giving a different mean difference in QALYs between groups across the two methods, suggesting they do not measure or value HRQoL in the same way as would be expected. This threatens the convergent validity of the VAS and SG methods to value change in HRQoL within the HF population. This lack of concordance between VAS, TTO and SG has been widely reported outside of HF [45]. Although most studies in the review used valuation tariffs that were derived using TTO methods, some used SG or VAS approaches to derive utility weights. The variation in methods for deriving QALYs identified in this review is therefore a concern and potentially threatens the consistency of the evidence on any decisions based on the findings of these evaluations. Nevertheless, the interventions reported generally appeared to be cost-effective when compared to specified decision-making thresholds for willingness to pay for a QALY gain. Therefore, it is possible that the variable statistical significance of the QALY gains identified in this review and the possible suboptimal sensitivity of some approaches to measuring and valuing HRQoL may not alter decision-making. However, reporting bias is also possible; interventions that are cost-effective are more likely to be published.

One possible solution to lack of sensitivity to change in generic PBM has been to measure change in HRQoL using a condition-specific measure and then to map these back to a generic PBM in order to generate utility weights [9, 46]. However, this may not maintain the sensitivity of the condition-specific measure if the generic PBM is not sensitive to these symptoms. This mapping approach was only observed within this review for the CARE-HF trial [11–13]. It has also been undertaken in several model-based studies in HF [39, 47, 48] but has received criticism regarding the potential error component in the algorithm used to map utility weights to the MLWHF [47]. Mapping is not a panacea and has been considered theoretically inferior [40, 46]. For example, the conversion of generic SF-12 scores into two different measures to derive QALYs in one study in this review [21] resulted in two different mean difference values between groups at follow-up, likely because the EQ-5D-3L does not contain a dimension for energy or vitality, leading to small and non-significant coefficients when mapped and potentially erroneous mapping values [49]. One study [19] within the review used a physician-reported measure, the NYHA to calculate QALYs by using a utility weighting from previous literature [32], which centres on domains of physical health and functional status as opposed to HRQoL. There is inherent uncertainty associated with both designating an NYHA class and the algorithm used to estimate utility weights, and it may not fully capture individuals’ HRQoL [50]. The three studies mapping MLWHF to EQ-5D-3L for the CARE-HF trial [11–13] identified in the review and several modelling studies [39, 51] all used the same algorithm reported by Calvert et al. [12]. It is unclear whether the method of estimation of weights on the basis of a mixed model relationship between the change in the EQ-5D-3L and MLWHF change is robust or not in capturing and modelling HRQoL changes. Standard guidelines exist for best practice methods in mapping studies [52–54], but the mapping algorithm utilised cannot be assessed against these as it has not been fully published or peer reviewed, only appearing in summary form in a cost utility analysis publication [12].

All of the studies identified in this review used the trial participants to describe their own health state for valuation, mostly via completion of a HRQoL instrument or a visual analogue scale (although, one study used physician assessment of NYHA class rather than the patient’s own perception of their health state as the basis for valuation). However, not all studies assigned the preferences of a general public sample to derive the valuation. Three studies used the preferences of patients with heart failure as the basis for the utility weights used to derive QALYs [15, 19, 25]. Arguments have been advanced in the literature both for and against the use of patient as opposed to public preferences for the valuation of health states [55–57]. However, consensus is generally aligned with the use of public preferences where the purpose of an evaluation is to inform resource allocation cross the health system [40], and NICE requires the use of public rather than patient preferences in their reference case [2].

### Limitations

Whilst we were inclusive in our approach to selecting studies, the diversity of the literature and necessity for narrow search terms may mean that some potentially relevant papers were missed. We reduced this risk by hand-searching identified papers. Publication bias is likely, but this is beyond the control of a systematic review. A language bias is also possible, as only published in English were retrieved. Our review only included trials which reported QALYs; thus, it does not present a complete picture of how HRQoL is measured or reported in heart failure. Trials that may have measured HRQoL but not derived QALYs, measured other aspects of the “patient journey” (e.g. the COMET study [58]) or studies reporting QALYs but principally using a modelling approach were excluded. This was a purposeful decision, since our focus was on how HRQoL was valued and QALYs were derived in primary research in heart failure.

Most participants included in this review were older men in NYHA class II or III, which is consistent with the majority of those enrolled in RCTs of HF. Limitations at the study and outcome level include the amount of missing HRQoL data, with some studies reporting 22–23% [16], 37% [30] and 12% [28] of participants with incomplete data. Approaches for dealing with missing data included the use of last-observation-carried-forward [11, 13, 22, 29] and imputation methods [20, 21, 23, 28], both of which have drawbacks in measuring HRQoL in the long term. In clinical trials where mortality is high and a utility weight of zero is assigned from the time of death, this may make a large contribution to the QALY value.

## Conclusions

Comparatively, few economic evaluations undertaken alongside clinical trials in patients with HF report QALY as an outcome measure. This is unfortunate given the importance of HRQoL (in addition to survival) as a treatment goal, both from a patient and health care professional’s perspective and for the determination of cost-effectiveness. This review suggests that the published evidence on cost-effectiveness that might underpin decisions regarding resource allocation for HF interventions is based on a variety of methodological approaches and usually relies on the sensitivity of generic measures. A review nearly 20 years ago suggested the optimal method of assessing HRQoL was a combination of both generic and condition-specific measures [8]. Findings suggest there has been no substantial progress in the most effective way to measure and value HRQoL for the purpose of deriving QALY outcomes in people with HF.

## Key points

- The accurate valuation of HRQoL is important to inform resource allocation decisions.
- We found few economic evaluations undertaken alongside clinical trials in heart failure have reported QALYs as an outcome.
- Trial-based economic evaluations have generally used generic measures of HRQoL to derive QALYs, but there is substantial variation in approach.
- Less than half the studies identified reported significant QALY gains between intervention groups
